# A cytogenetic study of couples with repeated spontaneous abortions

**DOI:** 10.4103/0256-4947.75785

**Published:** 2011

**Authors:** Shirin Niroumanesh, Parvin Mehdipour, Ali Farajpour, Soodabeh Darvish

**Affiliations:** From Mirza Koochak Khan Hospital, Medical Sciences/University of Tehran, Tehran, Iran

## Abstract

**BACKGROUND AND OBJECTIVE:**

The frequency of chromosomal aberrations in Iran is not definitely known. This study determined the frequency of chromosomal aberrations in a series of couples with two or more spontaneous abortions and compared the findings with that reported from other countries.

**METHODS:**

This was a descriptive study conducted on 100 couples with recurrent abortions. Both partners were karyotyped as part of the primary investigation. Other probable causes of abortion were also investigated.

**RESULTS:**

Chromosomal aberrations were found in 8 (8%) of the females and 5 (5%) of the males. The prevalence of chromosomal abnormalities was as follows: 4 (30.8%) balanced reciprocal translocations, 3 (23%) Robertsonian translocations, 3 (23%) pericentric inversions, 1 (7.7%) paracentric inversion, 1 (7.7%) chromosomal marker, and 1 (7.7%) polymorphism 9qh+.

**CONCLUSIONS:**

The pattern of chromosomal aberrations was similar to that reported in other studies, but the prevalence of chromosomal aberrations was higher.

Spontaneous pregnancy loss is the most common complication of pregnancy.^1^ Approximately 15% of all clinically recognized pregnancies are spontaneously aborted.^2^ Parental chromosomal abnormalities and antiphospholipid antibody syndrome are the only undisputed causes of spontaneous abortions. Other well-described causes are anatomic, endocrine, thrombotic and, possibly, some immunologic factors.^3^ It has been recommended that the standard investigation of such patients should include karyotyping of both parents for chromosomal aberrations.^4^ In the present study, we determined the prevalence of chromosomal anomalies in a series of Iranian couples with recurrent miscarriage and compared our findings with that reported from other countries.

## METHODS

A descriptive study was conducted on 100 couples (200 patients) referred to Mirza Kooochak Kahn hospital, Tehran, Iran, with two or more recurrent abortions before 20 weeks’ gestation within 1 year. Both partners were karyotyped as part of the primary investigation. Other probable causes of abortion were also investigated. For the chromosomal study, 0.5 mL of peripheral blood was incubated in a complete lymphocyte culture (20% fetal bovine serum with phytohemagglutinin and 1% Pen-Strep in 4.4% CO_2_ incubated at 37°C for 70 h). Cytogenetic analysis was performed by GTG-banding (G-banded using trypsin and Giemsa). Twenty metaphases were systemically studied and if any mosaicism was suspected, the number of analyzed metaphases was increased to fifty. At least three metaphases were photographed.

Chromosomal abnormalities were classified as major chromosomal abnormalities and minor. The aberrations consisted of reciprocal and Robertsonian translocations, inversions, deletions, sex chromosome aneuploidies, and mosaicism of either numerical or structural abnormalities. Regarding chromosome polymorphisms, only variants with particularly large or greatly reduced constitutive heterochromatin blocks and/or satellites were taken into account.

## RESULTS

A total of 100 couples with a history of repeated abortions were examined. The mean (SD) age of females was 27.2 (4.7) years and the mean age of the males was 31.3 (4.6) years. In addition to the history of repeated abortion, some had a history of stillbirth or of having a child with malformative syndrome or mental retardation (e.g., Down syndrome). Of the 396 pregnancies in 100 couples, 366 (92.4%) ended in abortion and 30 (7.6%) ended in delivery (including stillbirth, Down syndrome, and a healthy infant). The number of abortions ranged from 2 to 10 with a mean of 2.7 (1.6). The prevalence of presumptive causes of repeated abortions among couples in this study was as follows: anatomic abnormality 22%, hormonal defects 5%, immunologic causes 6%, infectious causes 19%, chromosomal aberrations 12%, and unknown causes 36%.

Eight females (8%) and five males (5%) were found to have abnormal karyotypes. There was a double abnormality, inv(9)(q22;q13), in both the male and the female in one couple (**Table 1**). Major chromosomal abnormalities were identified in nine (69.2%) patients with abnormal karyotypes and chromosomal variants in four (30.8%). These abnormalities included four (30.8%) balanced reciprocal translocations (two male and two female), three (23%) Robertsonian translocations (one male and two females), three (23%) pericentric inversions (two male and one female), one (7.7%) paracentric inversion (a female), one (7.7%) chromosomal marker (a female), and one (7.7%) polymorphism 9qh+ (a female). All Robertsonian translocations were D/G. (D/G occurs between one of chromosomes 13-15). Among cases with pericentric inversions, one couple had pericentric inversion 9 and one male had pericentric inversion 7. Paracentric inversion was found in one female at chromosome 16.

**Table 1:** Cytogenic study, number of abortions, and parental age in cases with abnormal karyotypes.

Karyotypes	Sex	Age	First-trimester abortions	Second-trimester abortions	Offspring or stillbirths
46,XY, t(2;12)(q31;q24)	M	37	3	0	1
46,XX, t(9;13)(p11;q33)	F	28	3	1	0
46,XX, t(3;8)(q21;q24)	F	30	3	2	2
46,XY, t(7;13)(q22;q24)	M	34	3	1	1
45,XX,-14,-21,t(14q;21q)	F	30	2	0	1
45,XY,-13,-22,t(13q;22q)	M	28	4	1	0
45,XX,-15,-21,t(15q;21q)	F	26	3	1	1
46,XX, inv (9)(q22;q13)*	F	26	0	4	0
46,XY, inv (9)(q22;q13)*	M	26	0	4	0
46,XY, inv (7)	M	27	1	3	0
46,XX, inv (16)(q11;q22)	F	30	0	5	0
46,XX, Inc Frag/ 46,XX	F	27	1	0	0
46,XX, 9qh+	F	27	7	0	1

t: translocation; inv: inversion.

* One couple with a similar chromosomal aberration.

Of a total of 59 pregnancies in 13 patients with chromosomal aberrations, 7 (13%) ended in birth, with 4 (6.8%) healthy infants, 2 (3.4%) stillbirths, and 1 (1.7%) infant with Down syndrome. The other 52 pregnancies ended in abortions: 30 were first abortions and 22 were second abortions.

## DISCUSSION

The prevalence of parental chromosomal aberrations among couples in this study was 12%, which is greater than that reported by other authors; nine were major chromosomal abnormalities and four were minor aberrations. However, the pattern of chromosomal abnormalities is similar to that seen in previous studies.^5^–^7^ The high prevalence may be because our cases were selected from among subjects who had had two or more spontaneous abortions, whereas most of the other studies included subjects with three or more repeated miscarriages. We determined that eight women and five men had chromosomal aberrations. This female-to-male ratio (1.6/1) was not different from that found in most other studies.^5^,^8^ A likely explanation is that chromosomal aberrations in male carriers may cause severe meiotic disturbances and spermatogenic arrest. Some chromosomal abnormalities (such as Robertsonian translocations) that are compatible with fertility in women may be associated with sterility in men.^3^,^9^ As has also been reported in other studies,^9^ reciprocal translocations were the most frequent balanced chromosomal anomalies detected in this study. However, the most prevalent type of translocations in our study was D/G, whereas D/D translocation is the most common one reported in the literature.^3^

Pericentric inversions were found in three patients: one was in chromosome 7 and two in chromosome 9. A paracentric inversion was determined in one female in chromosome 16. All of these patients had history of repeated second-trimester abortions. Outcomes of paracentric inversions are more harmful than that of pericentric inversions. Indeed, the risk of chromosomal imbalance in pericentric inversions depends on the length of the inverted segment.^10^

In the present study there was one chromosomal marker (mosaicism type) in a female with two repeated abortions. Probably the fragment of chromosomal marker included important genes and therefore any defect increased the risk of having an unbalanced zygote, and a resultant abortion. There was also one polymorphism in the 9qh+ region of a female who had a history of eight abortions and one live birth.

This study provides the first results from Iran on the prevalence of chromosomal aberrations in couples with history of repeated abortions. The pattern of chromosomal aberrations is similar to that seen in other studies, but the prevalence is higher in our study. In view of these results, we recommend that patients who have had two or more miscarriages (and not only three or more) be advised to have their karyotypes checked.
